# Male Attractiveness Is Influenced by UV Wavelengths in a Newt Species but Not in Its Close Relative

**DOI:** 10.1371/journal.pone.0030391

**Published:** 2012-01-17

**Authors:** Jean Secondi, Virginie Lepetz, Marc Théry

**Affiliations:** 1 GECCO, Group Ecology and Conservation of Vertebrates, University of Angers, Angers, France; 2 UMR 7179, Centre National de la Recherche Scientifique/Muséum National d'Histoire Naturelle, Brunoy, France; University of Jyväskylä, Finland

## Abstract

**Background:**

Functional communication in the UV range has been reported in Invertebrates and all major groups of Vertebrates but Amphibians. Although perception in this wavelength range has been shown in a few species, UV signalling has not been demonstrated in this group. One reason may be that in lentic freshwater habitats, litter decomposition generates dissolved organic carbon that absorbs UV radiation and thus hinders its use for visual signalling. We tested the effect of male UV characteristics on female sexual preference in two newt species that experience contrasting levels of UV water transmission when breeding.

**Methodology/Principal Findings:**

We analysed water spectral characteristics of a sample of breeding ponds in both species. We quantified male ventral coloration and measured male attractiveness under two lighting conditions (UV present, UV absent) using a no-choice female preference design. UV transmission was higher in *Lissotriton vulgaris* breeding sites. Male UV patterns also differed between experimental males of the two species. We observed a first common peak around 333 nm, higher in *L. vulgaris*, and a second peak around 397 nm, more frequent and higher in *L. helveticus*. Male attractiveness was significantly reduced in *L. vulgaris* when UV was not available but not in *L. helveticus*. Male attractiveness depended on the hue of the first UV peak in *L. vulgaris*.

**Conclusion/Significance:**

Our study is the first report of functional UV-based communication in Amphibians. Interestingly, male spectral characteristics and female preferences were consistent with the differences in habitat observed between the two species as *L. helveticus* often breeds in ponds containing more UV blocking compounds. We discuss the three hypotheses proposed so far for UV signalling in animals (enhanced signal detectability, private communication channel, indicator of individual quality).

## Introduction

Ultraviolet radiation is perceived by several groups of Invertebrates and Vertebrates. It is used in functions as diverse as orientation ([1]; [2]), regulation of circadian rhythms ([3]), foraging of predators ([4]; [5]) or frugivorous species ([6]; [7]; [8]), prey attaction ([9]), crypsis ([10], [11]), and intraspecific communication ([12]; ([3]). UV signals are used in food provisioning of offspring by parents ([13]) and in both intrasexual ([14]; [15]) and intersexual communication. UV-based mate assessment has been described in arthropods ([16], [17]), birds ([18],[19]; [20]; [21]), fish ([22]; [23]; [24]; [25]) and reptiles ([26]; [27]; [28]; [29]), but remains unstudied in Amphibians.

Three main hypotheses account for the specific use of UV wavelengths in sexual communication. (i) Enhanced detectability – UV signals offer a larger contrast with the background than other visible signals would and thus enhance signalling efficiency ([21]; [30]; [27]). (ii) A private communication channel – Signalling costs may be reduced if signals are not perceived by predators, i.e. if they lack UV sensitive photoreceptors ([25], [31]). (iii) Indicator of mate quality – UV-based signals provide information about the quality of a potential partner ([18]; [32]) or its parasitic status ([33]) and may influence assortative mating ([21]; [34]). UV signalling may have effects at the species level on the mate selection process (sexual selection). It also has the potential to modulate interspecific interactions such as predator-prey relationships ([25]), interspecific competition ([27]) and hybridization ([35]).

Surprisingly, Amphibians remain the last major group of vertebrates where UV communication has not been observed. In spite of the bright colorations and complex displays of some species ([36]; [37]; [38]), little is still known about their visual perception and the function of colour signals compared to other Vertebrates ([39]; [40]). More generally, colour perception and its variation in relation with ecology are still poorly understood in this group. We have some hints about the use of UV communication though. Oil droplets that can filter out UV radiations are found in anuran but not in urodeles and caecilians ([41]). More direct evidence of UV perception comes from urodeles (but see [42]; [43] for anurans) where sensitivity peaks in the UV range have been reported ([44]; [45]; [46]; [47]). However, if studies demonstrated the ability to perceive UV radiations, they did not investigate whether UV perception was associated with any function. So far, the stronger evidence comes from the observed increase in UV colour intensity during the breeding season in a frog ([48]).

The role of colour signals in mate choice is suspected in newts ([49]). The Smooth newt *Lissotriton vulgaris* and the Palmate newt *L. helveticus* both display bright yellow-orange coloration on their underparts but UV has not been investigated. We report here the presence of UV components in the ventral reflectance spectra of these two urodeles. If UV wavelengths are used for signalling in newts, male attractiveness should drop when these are not available in the medium. We tested this hypothesis using a no-choice preference design. The two species exhibit different habitat preferences. *L. vulgaris* preferentially uses open habitat ponds whereas *L. helveticus* also breeds in forest ponds ([50]). Litter decomposition in forest ponds generates high concentrations of dissolved organic carbon (DOC) that strongly absorbs UV radiations ([51]). Levels of UV radiations should therefore be in average lower in *L. helveticus* breeding habitats than in *L. vulgaris* breeding habitats. Accordingly, we predict that UV components may contribute to male attractiveness only *in L. vulgaris*.

## Methods

### Habitat and newt spectral characteristics

Capture and experiments have carried accordingly to Permit delivered by Préfecture du Maine et Loire et Préfecture de Loire Atlantique in 2009 and 2010. Permit ID are the following: n°01/2009 and n°12/2010.

Between 2007 and 2010 we collected water samples from 50 ponds in the study area. One sample was taken per pond as DOC concentrations are not expected to vary in closed water bodies. Samples were collected about 30 cm under the surface prior any action in the pond. We are not aware of studies reporting the distribution of courting depths in different habitats but sampling was carried out within the range at which newt court (J.S. pers. obs.). Thirty-five ponds were in allotopy (*L. helveticus* only) among which 13 were forest ponds surrounded by ligneous vegetation. The remaining 15 ponds were in syntopy in more open habitat. We never found *L. vulgaris* alone. Subjects were anaesthetized by immersion in 0.2 g/l Tricaine methanesulfonate (MS222) before measurements.

We measured transmission spectra of water samples using a UNICAM, UV-Visible 2 spectrophotometer and the software Vision. In order to compare water transmission spectra of forest *L. helvecticus*, open *L. helvecticus* and *L. vulgaris* ponds we calculated the average transmission in two wavelength ranges 300–420 nm (UV) and 420–700 nm. Samples have been stored at 4°C for a maximal duration of 3 weeks. Storing conditions are not expected to alter optical water characteristics. Concentration in compounds like DOC will remain stable over such a duration.

We measured the reflectance spectra *R*(λ) between 300–700 nm of ventral body for all experimental males. We used a spectrometer (Ocean Optics S2000), a deuterium-halogen light source (Ocean Optics DH-2000), and a coaxial optic fibre (Avantes FCR-7UV200-2-45-ME). We took two measurements and selected the one that showed the highest UV brightness. Analyses were carried out with AVICOL v.3 ([52]). In the UV range (300–420 nm), we computed brightness and peak wavelength, i.e. the wavelength of maximal reflectance. In the visible range (420–700 nm), we calculated brightness and hue, i.e. the wavelength of the steepest slope of the reflectance curve. We also computed chroma in the UV and in the visible range using the maxminchroma option (Maxmin chroma = Abs((Rmax-Rmin)/Rav), where Rmax is the maximal reflectance, Rmin the minimal reflectance, and Rav the average reflectance. We did not use vision models. They provide more accurate measurements of how colours are perceived but they require a priori knowledge of cone sensitivities. These have been determined in one urodele species only the Tiger salamander *Ambystoma tigrinum* and there is consequently no evidence that sensitivities are constant in this group.

### Experiment– Effect of UV on male attractiveness

#### Samples and housing conditions

We captured 50 *L. helveticus* and 50 *L. vulgaris*, 25 of each sex, in two syntopic ponds in an area of broad sympatry between the two species. Syntopic sites are sites within the zone of sympatry where the two species physically co-occur. The ponds were located west of Angers (France) in the Loire river floodplain and surrounded by open habitat. *L. helveticus* individuals were caught in one pond on March 31^st^ 2010 and *L. vulgaris* individuals in the other pond on April 1^st^ 2010. Tests were carried out between April 6–10. Typically the peak of the breeding season in the study area is March-April. Subjects were housed singly in indoor aquaria (length 51.5 cm×width 34 cm×height 32.5 cm) filled with 8 litres of aged tap water and kept at a room temperature of 15°C.

#### Behavioural tests

We used a no-choice design to test the effect of UV on intraspecific male attractiveness. The experimental apparatus was a glass aquarium (40 cm length×20 cm width×15 cm height) filled with 10 cm of clear aged tap water. One male was placed in a box, 6.5 cm long, which was made of black plastic and inserted at one end of the aquarium. One female was placed on the other side of the aquarium. We defined a preference zone that extended 5 cm away from the box. Because strong direct light may disturb newt motor activity, we shaded the whole apparatus but the male box. A window was additionally cut on each side of the box to allow lateral light transmission and limit shadows cast on ventral body parts. The front panel of the box was made of a filter inserted into a removable frame of the same black plastic as the rest of the box. The sides of the apparatus and its top (except the male box) were blocked with black plastic plates.

We measured female response to the same male under two lighting conditions: full spectrum with UV (UV+) and without UV (UV−). This matched design ensured that UV+ and UV− stimuli only differed by the amount of UV radiation they reflected ([28]). We used a UV filter for the UV− treatment (Eurofilter 226, transmission 3% at 360 nm) and a neutral grey filter for the UV+ treatment (Eurofilter 298) in order to compensate for the loss of total brightness caused by the filtering of the UV band in the UV− treatment [53]. Irradiance spectra available to subjects in the two treatments are given as supporting information (Figure S1). We combined two fluorescent tubes to generate a full spectrum (daylight 20W Repti-Glo 2.0, UV-rich 20 W Repti-Glo 5.0). According to the manufacturer guideline, tubes were placed close to the water surface (20 cm). Test periods for each treatment lasted 10 minutes. Females were kept in the distal end of the aquarium by a mesh wire fence for two minutes prior to each test period. For each period, we measured the association time with the male, i.e. time spent in the preference zone. Treatment order was alternated between sessions, and each pair was tested in a single session. Chemical exchanges between individuals were allowed to enhance female motivation. Individuals were released at the capture site after the experiment. Lighting condition could potentially affect pheromone release. Because males were kept under constant lighting conditions, unlike females, we assume that no changes in male pheromone production occurred during a test session.

Male brightness in the UV and visible range was modified between treatments because of filters. We checked that the difference in female behaviour between treatments (UV, grey) was not altered by the difference in brightness. To estimate how much male brightness was available to females, we multiplied each male reflectance spectrum by the ambient light irradiance, and the UV or and neutral grey filter transmission spectrum according to treatment. In each treatment all females spent time in the preference zone, a few centimetres away from the male, and many of them were separated from the male only by the filter thickness. Because interindividual distances were so short, we got a reasonable estimate of available brightness to females in each treatment.

### Statistical analyses

Spectral variables were not normally distributed and could not be transformed. Thus, we tested spectral differences of water between species and habitat using Kruskal-Wallis tests. Pairwise comparisons were tested using Mann-Whitney tests and Bonferroni corrections. We also analyzed interspecific differences in male colour variables using Mann-Whitney tests. Differences of female response between treatments (UV+ minus UV- treatment) were normally distributed for both species and were tested using paired t-tests. Relationships between spectral characteristics and female responses were analysed using linear regression. All analyses were carried out using R 2.10 ([54]).

## Results

### Spectral characteristics of habitat and males

**Figure 1:**
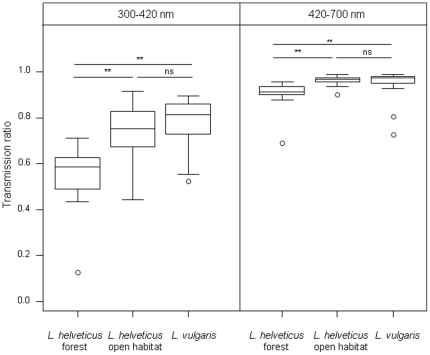
Terrestrial habitat and light transmission in water. Transmission spectra of water averaged over the 300–420 nm (UV) and 420–700 nm. Asterisks indicate p<0.01 with Mann-Whitney tests after checking for global significant differences between group using Kruskal-Wallis tests. ns = non significant, ** p<0.001.

Radiations were more absorbed in *L. helveticus* breeding ponds (syntopic and allotopic) than in *L. vulgaris* breeding ponds (syntopic only) in the UV range (W = 147, *P* = 0.015) but they were not in the 420–700 nm range (W = 190, *P* = 0.127). Interspecific differences were caused by forest ponds where only *L. helveticus* breeds. Light transmission in the three categories of ponds (*L. vulgaris*, forest *L. helveticus*, and open habitat *L. helveticus*) differed significantly in the UV range (KW = 19.589, df = 2, *P*<0.0001). UV radiations were less transmitted in forested *L. helveticus* ponds than in open habitat *L. helveticus* ponds (W = 27.5, *P*<0.0001) or *L. vulgaris* ponds (W = 16, *P* = 0.0002). Transmission did not differ between *L. vulgaris* and open habitat *L. helveticus* ponds (W = 131, *P* = 0.304) (Figure 1). The pattern was similar in the 420–700 nm range. The three categories of sites differed significantly (KW = 16.132, df = 2, *P* = 0.0003). Wavelengths were more absorbed in forest *L. helveticus* ponds than in open habitat *L. helveticus* ponds (W = 26.5, *P*<0.0001) or *L. vulgaris* ponds (W = 33, *P* = 0.0032). Average transmission did not differ between the last two types of ponds (W = 157, *P* = 0.819) (Figure 1). Most forest ponds were located on the plateaus of the Loire river. The studies we have been conducting since 2004 have shown that *L. vulgaris* does not occur there [50]. In floodplain sites, we actively searched the two species. We cannot definitively discard the possibility that we missed some *L. vulgaris* individuals but their number would have been very low and probably insufficient to consider as a sustainable breeding population. Nevertheless, we believe that the risk of omission, i.e. the wrong assignment of a pond to a category (allotopic/syntopic), is low and it does not alter our conclusions as interspecific differences in water spectra occurred between forest and open habitat ponds.

**Figure 2:**
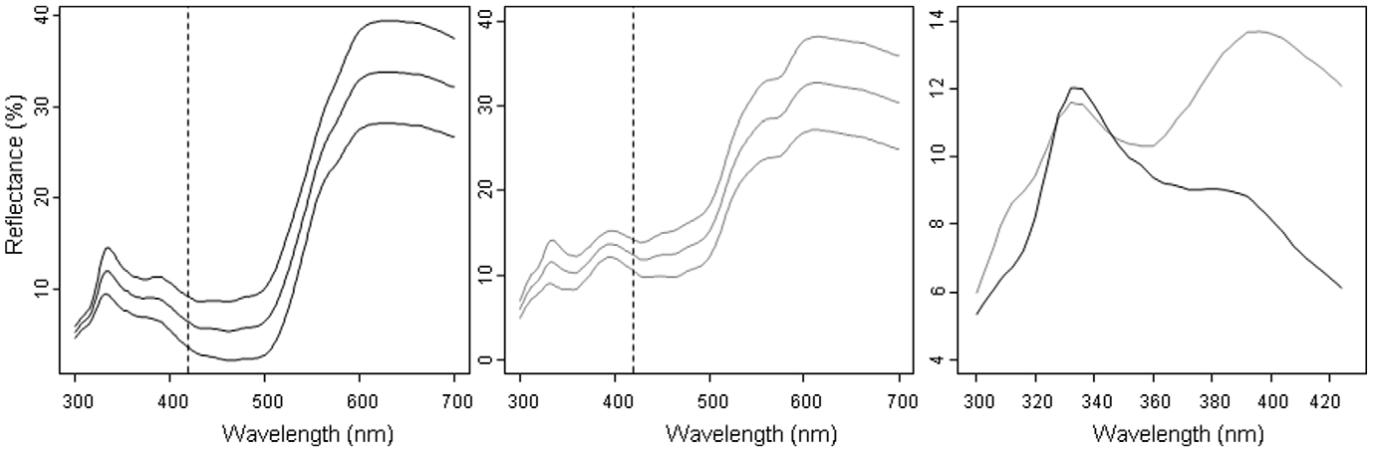
Ventral reflectance spectra. Mean (±SD) ventral spectra of *Lissotriton vulgaris* (left) and *L. helveticus* (center) tested in the experiment. Close-up on the UV range that shows the two peaks in *Lissotriton vulgaris* (black line) and *L. helveticus* (grey line) in the UV range. The dotted lines indicate the limit of the UV range.

The reflectance spectra of the two species differed for most variables. Brightness was lower in *L. vulgaris* than in *L. helveticus* both in the UV and visible ranges (Table 1). Both *L. vulgaris* and *L. helveticus* showed a single peak in the UV range around 330 nm. Figure 2 shows the mean reflectance spectra of experimental males. The close-up (right panel) illustrates the contrasting UV pattern between the two species, each displaying a dominant peak at different wavelengths. The wavelength of the shorter UV peak was the only variable not to show significant differences between the two species (Table 1). The wavelength of the longer UV peak was significantly higher than that of the shorter UV peak in *L. helveticus* (Mann-Whitney test: *n* = 49, W = 0, *P* = 2.2e-16). Hue was higher, i.e. more orange, in *L. vulgaris*. Finally, UV chroma was lower in *L. vulgaris* than in *L. helveticus* whereas it was much higher in the visible range (Table 1).

**Table 1:** Colour variables of the ventral body measured in 49 male *Lissotriton vulgaris* and 49 male *L. helveticus*.

	*L. vulgaris*			*L. helveticus*				
Colour variable	*n*	Mean	SD	*n*	Mean	SD	*W*	*P*
Mean brightness (300–420 nm)	49	8.81	1.89	49	11.20	1.48	2087	<0.0001*
Mean brightness (420–700 nm)	49	20.83	3.39	49	24.24	3.57	1843	<0.0001*
Shorter UV peak wavelength	49	334	1.89	49	333	2.96	1098.5	0.465
Longer UV peak wavelength	48	NA	NA	49	397	7.84	NA	NA
Hue (420–700 nm)	49	539	12.44	49	516	3.90	163	<0.0001*
UV Chroma (300–420 nm)	49	0.15	0.02	49	0.17	0.02	1628	0.002*
Chroma (420–700 nm)	49	0.80	0.08	49	0.58	0.11	95	<0.0001*

Figures in bold indicate significant *P*-values for α = 0.05 and asterisks significant p-values after Bonferroni corrections (α = 0.008).

### Effect of UV on male attractiveness

**Figure 3:**
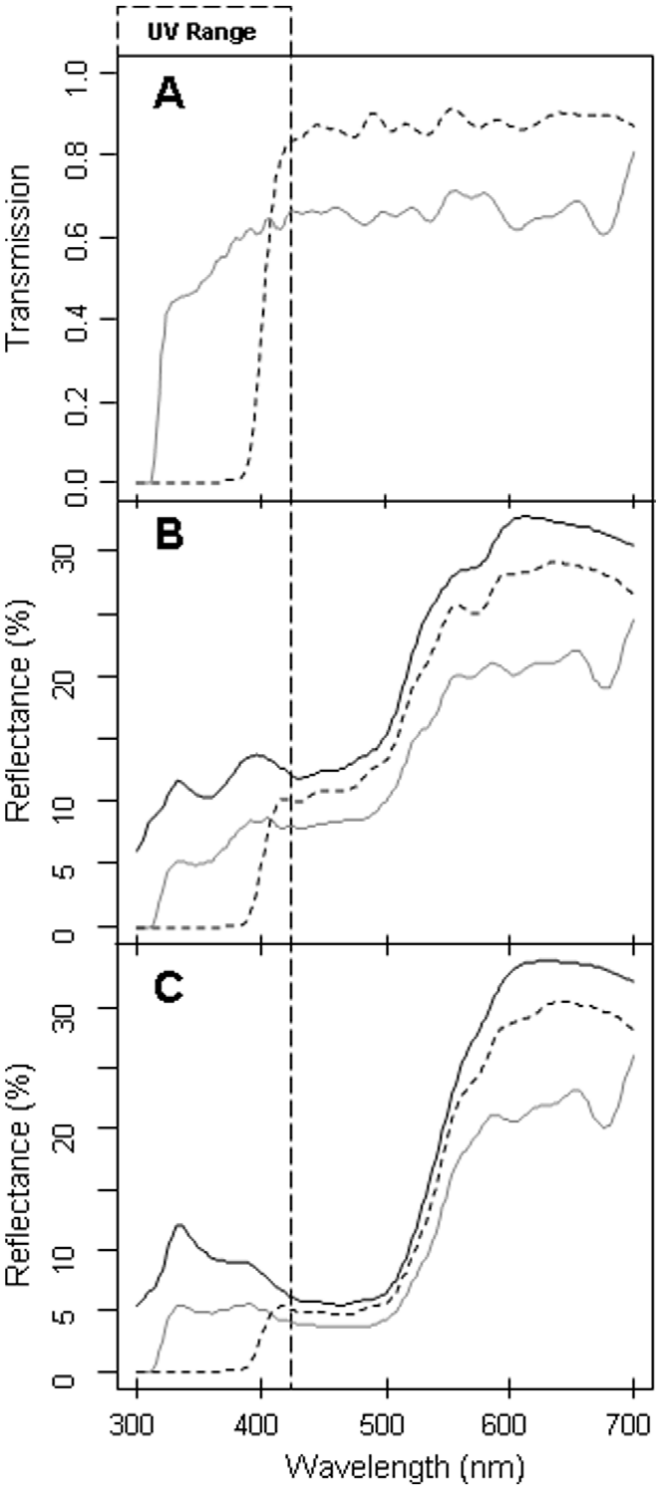
Effect of filters on male ventral colour. (A) Transmission of filters used in the UV+ (solid line) and UV− (dotted line) treatments. The two lower panels show the mean ventral spectra (black solid lines) and the products of transmission and reflectance of ventral mean specta for the UV+ (grey line) and the UV− filter (dotted line) for *Lissotriton helveticus* (B) and *L. vulgaris* (C).

**Figure 4:**
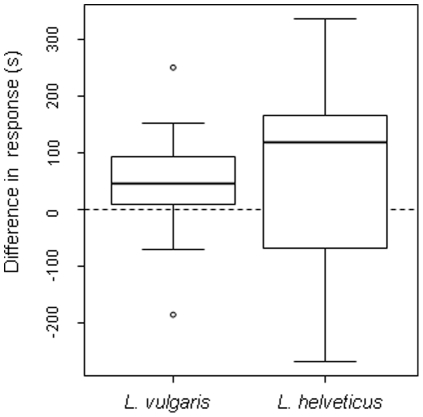
Effect of UV availability on female preference. Difference between treatments (UV+ and UV−) in time spent close to conspecific males by *Lissotriton helveticus* and *L. vulgaris* females.

**Figure 5:**
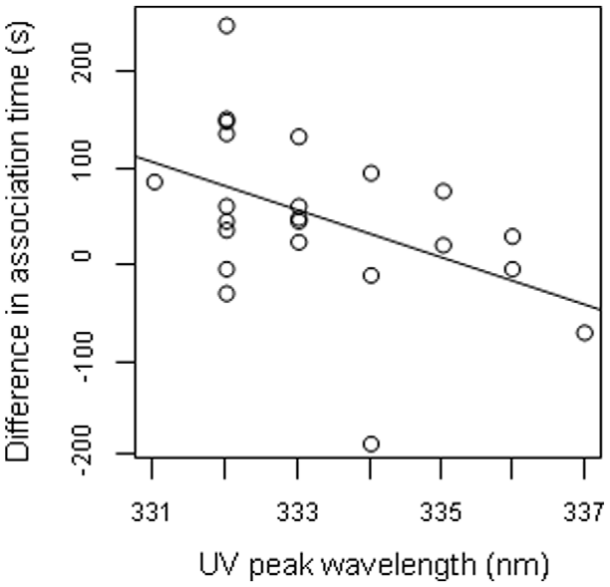
Male UV signal and female preference. Effect of UV peak wavelength on the difference in response between treatments (UV+ and −UV−) in females *Lissotriton vulgaris*.

Figure 3 shows the effect of filters used in the UV+ and UV− treatments in the two species. In female *L. vulgaris*, association time was higher during the UV+ treatment than during the UV- treatment (UV+ = 257.48s±114.19s, UV− = 206.83s±110.89s, *n* = 23, t = 2.781, *P* = 0.011) (Figure 4). The intensity of female association, i.e. the time spent close to the male, depended on male UV characteristics as the difference in response between UV+ and UV− was negatively related to the wavelength of the lower UV peak (slope = −24.61±10.62 SE, df = 21, F = −2.318, *P* = 0.031) (Figure 5). The shorter the wavelength was, the higher was the drop in female response during the UV− treatment. Neither hue in the visible range, or chroma in the UV and visible ranges significantly affected female response (all *P*>0.18). Similarly, the difference of brightness available to females did not affect the difference of female response between treatments in the UV range (F_1.21_ = 0.207, *P* = 0.653) or the 420–700 nm range (F_1.21_ = 0.717, *P* = 0.407).

Female *L. helveticus* tended to spend more time close to the male when UV light was available but the difference between treatments was not significant (UV+ = 343.12 s±284.2 s, UV− = 143.32s±155.7 s, *n* = 25, t = 1.702, *P* = 0.102). Overall, responses were more variable in this species (Figure 4). We observed no significant relationships between the difference in association time and the wavelengths of the shorter and longer UV peaks, and chroma in the UV or visible ranges (all *P*>0.255). The difference of available brightness did not affect the difference of female response between treatments in the UV range *(*F_1,23_ = 1.27, *P* = 0.271) and in the 420–700 nm range (F_1,23_ = 0.0003, *P* = 0.986).

## Discussion

### Effect of UV on male attractiveness

In *L. vulgaris*, male attractiveness was significantly reduced when UV radiations were filtered out. The loss of attractiveness also depended on the UV peak wavelength as the reduction of female response was larger for stimulus males with shorter wavelengths peaks. This means that UV-based attractiveness did not entirely depend on signal intensity. Females also assessed peak wavelength that, unlike brightness, is not altered by light scattering ([55]). Although the range over which that effect was observed is strikingly narrow, only 6 nanometres in the study population, it is still biologically relevant.

In *L. helveticus*, we observed no relationships between male colour and the difference in female response between treatments. We did not detect a significant reduction of male attractiveness when UV radiations were filtered out. However, the trend was the same as in the other species. Because the dominant peak is at the upper limit of the UV range in this species, it is possible that we did not block satisfactorily all UV components. This peak should be less degraded by DOC ([51]) and be more effective for communication in *L. helveticus* habitats. Thus, at this point we cannot rule out the hypothesis of UV perception in *L. helveticus*. Note that in both species, the difference in female response could not be attributed to differences in male brightness between treatments.

We tested populations from two open habitat ponds which allows conservative conclusions about UV-based communication. We found no strong evidence in *L. helveticus*. Thus, there is no reason to believe that newts would be more prone to use UVs in forest ponds where these radiations are drastically filtered out by water. Nevertheless, UV-based communication could be impaired in other parts of the *L. vulgaris* range if populations were to breed in low UV-transmission ponds. This hypothesis remains to be examined.

### Function of UV signals

Enhanced signal detectability, a private communication channel, and quality indicator are the three functions proposed for UV signals. Shorter wavelengths are more scattered by water molecules and suspended particles than longer wavelengths. Scattering generates a ‘veiling light’ that reduces signal discriminability ([55]). In addition, in lentic water bodies DOC and suspended particles cause excess attenuation of UV wavelengths ([51]). Scattering and absorption by DOC reduce the depth and distance ranges over which UV transmission is possible. Authors ([25]) suggested that UV signals were better designed for short range communication in aquatic organisms. Consistently, partners get close during courtship in newts which allows the assessment of ventral visual cues ([36]).

The private communication channel hypothesis has received limited support for terrestrial vertebrates so far ([56]). Aquatic species might be better candidates because fishes, a major group of predators, exhibit large interspecific variation of UV sensitivity, and their habitats vary widely in the relative amounts of UV present ([39];[25]). Newts usually breed in fishless habitats so that fishes may not drive the evolution of a private channel but birds or insects like *Dysticus* species may use UV to locate newts when these are respectively surfacing and swimming. If UV signals do not avoid detection by predators, they could prevent eavesdropping from conspecifics ([57]), as suggested for fish ([39]), and reduce the risk of interference with other males. Here, a private communication channel would rather contribute to regulate intraspecific than interspecific interactions.

Finally, UV signals may advertise male quality intra- or intersexually. Male interference is moderate in these species and fighting is not observed. Nevertheless, peak wavelength may constitute a dominance cue that regulates agonistic interactions and prevents fighting as observed in a lizard ([15]). Higher crest in *L. vulgaris* ([58]) or longer caudal filaments in *L. helveticus* ([59]) are assessed by females. UV signals may be correlated with such attributes. Alternatively, UV signals might be assessed for species recognition. The longer UV peak in *L. helveticus* may be used to that effect, which is relevant for species in which hybridization frequency has been recently re-evaluated ([60]).

### Signal and preference tuning to habitat support the sensory drive hypothesis

Habitat selection determines the constraints and opportunities for signalling ([61], [62]). In this regard, the two newt species present an interesting model as they exhibit overlapping ecological requirements for their breeding habitat. The ubiquitous *L. helveticus* commonly breeds in forest ponds whereas *L. vulgaris* prefers non-forested habitats. In the study area, *L. vulgaris* habitat is strictly nested within *L. helveticus* habitat so that allotopic sites are not known for *L. vulgaris*.

The discrepancy in their terrestrial habitat was reflected in the visual environment of the two species. The range and variability of light transmission conditions were greater for *L. helveticus*. The difference mostly occurred in the UV range. Differences, albeit significant, where much more reduced in the 420–700 nm range. As expected UV radiations were more absorbed in forest ponds exploited by *L. helveticus*, probably because of the higher DOC concentrations resulting from litter degradation. Thus, water spectral characteristics reflected the difference in habitat selection observed in the two species. Accordingly, signal characteristics and female preference were consistent with the UV radiation levels in the breeding habitats of each species. Although more populations need to be tested to confirm that pattern, that first study strongly suggests that the visual communication system of each species is tuned to its environment, and thus supports the sensory drive hypothesis ([63]).

To our knowledge, this study is the first report of UV signalling in Amphibians. We did not demonstrate the actual function of the UV signal but we clearly showed that it influenced male attractiveness. It is noticeable that evidence comes from *L. vulgaris*, the species breeding in habitats that offer the best conditions of signal transmission. Interestingly, UV radiations are almost unequivocally viewed as a threat to Amphibians because of their deleterious effect to larval development and on adults ([64]). The present study demonstrates that Amphibians can exploit UV light for signalling too.

## Supporting Information

Figure S1 **Effect of filters on irradiance spectra.** Irradiance spectra for the UV+ (grey lines) and UV− treatments (black lines). Irradiance is the product of the irradiance produced by the light tube times the transmittance of the filter.
(TIF)
